# Phenotypic variability in two female siblings with oocyte maturation arrest due to a *TUBB8* variant

**DOI:** 10.1186/s12920-023-01712-7

**Published:** 2023-10-30

**Authors:** Qian Dou, HongEn Xu, LiYing Ma, Li Tan, WenXue Tang

**Affiliations:** 1 Reproductive Medicine Center, The Second Affiliated Hospital of Zhengzhou University, Zhengzhou, China; 2 Precision Medicine Center, Academy of Medical Science, Zhengzhou University, Zhengzhou, China

**Keywords:** *TUBB8*, Oocyte maturation arrest, Embryo development arrest, Phenotypic variability, Female infertility

## Abstract

Tubulin beta-8 (*TUBB8*) is expressed exclusively in the oocyte and early embryo, encoding a beta-tubulin polypeptide that participates in the assembly of microtubules. *TUBB8* was first attributed to being responsible for oocyte MI arrest. Further studies have demonstrated that patients with different pathogenic variants have variable phenotypes. We report a *TUBB8* variant (c.10 A > C) in two siblings who presented different clinical features of primary infertility. The younger sister showed severe oocyte maturation arrest with abnormal morphology, whereas a few mature oocytes and zygotes could be retrieved from the older sister, but no embryo was available for transfer. This variant was previously reported without in vitro functional assays. In the present study, RT‒qPCR and western blot analyses revealed that c.10 A > C reduces *TUBB8* mRNA and protein levels; however, immunofluorescence demonstrated that this variant does not change the localization of the protein. These findings confirm the pathogenicity of the c.10 A > C variant and support the relationship between the variant and phenotype in the patients.

**Supplementary Information:**

The online version contains supplementary material available at 10.1186/s12920-023-01712-7.

## Background

Recently, assisted reproductive technology (ART) has been widely applied as a treatment for infertility [1], but only with a success rate of approximately 30–40% [2]. Embryonic development and endometrial receptivity are key factors for a successful pregnancy. The quality of the oocyte determines embryo development and diminishes with increasing maternal age [3, 4]. However, females of optimal age have experienced unexplained infertility or repeated failure of in vitro fertilization (IVF) treatments. The underlying cause may be oocyte or sperm related [5]. With the rapid advancement of next-generation sequencing technologies, more crucial genes for oocyte maturation, fertilization, and early embryo development have been identified [2]. Any defects in these genes might explain the embryonic failure of IVF cycles [6].

Mammalian oocytes mature through meiosis to prepare for fertilization [7]. After fertilization, the zygote undergoes multiple mitosis rounds, forming the early embryo. The spindle is a crucial structure in both meiosis and mitosis for segregating chromosomes and is composed of microtubules (MTs) and many types of molecular motors [8, 9]. Normal spindle structures and functional dynamics are indispensable for meiosis and mitosis progression [10]. MTs are hollow tubes assembled from α/β-tubulin heterodimers [11, 12]. The human β-tubulin family has ten isotypes [11], and *TUBB8* is the only isotype specifically expressed in human oocytes and early embryos, which indicates that it plays a role in spindle assembly in humans [13].

In 2016, *TUBB8* was first described and identified as responsible for oocyte MI arrest [13]. Further studies have expanded the mutational spectrum of *TUBB8* in human oocyte development, fertilization, and early embryo development [14–25]. Screening *TUBB8* pathogenic variants has potential value for genetic diagnoses of infertile couples for whom ART treatment has recurrently failed [2, 19].

Pathogenic variants of *TUBB8* result in at least five phenotypes: oocyte MI arrest, failed fertilized oocytes, embryo failure to cleave, early embryonic development arrest, and embryo implantation failure [13–25]. However, patients with the same variant in different reports showed phenotypic variability [15, 19, 23]. In this study, a missense variant (c.10 A > C, p.I4L) of *TUBB8* was identified in two siblings with different clinical features of primary infertility. The phenotype of oocytes and embryos described is mainly based on optical microscope observations, but the spindle structure cannot be directly observed. The heterogeneity of the phenotype increases the difficulty of clinical diagnosis. The older sister underwent three cycles of ART treatment, with failure. It was not until the younger sister’s oocytes presented serious abnormal morphology that it was considered that the older sister also had genetic alterations. Although the variant p.I4L has been reported [15, 19, 23], no causative relationship has been established between the variant and female infertility. In this study, we demonstrate that this variant leads to reduced *TUBB8* mRNA and protein levels, but with no change in protein localization. The results provide evidence of the pathogenicity of the variant and a reference for clinical decision-making.

## Materials and methods

### Human subjects

The patients were recruited from the Reproductive Medicine Center, the Second Affiliated Hospital of Zhengzhou University, China. Clinical profiles and embryo laboratory data were obtained from the electronic medical records system of the hospital.

### Ethical approval

All studies on human subjects were approved by the ethics committee of the Second Affiliated Hospital of Zhengzhou University (reference No. 2,022,373). The patients signed written informed consent.

### Clinical protocol, in vitro fertilization, and embryo culture

The patients were treated with controlled ovarian hyperstimulation (COH). The initial gonadotrophin dose ranged from 112.5 ~ 300 IU/day and was later adjusted based on follicular growth rate and hormone levels. When 1 ~ 2 dominant follicles reached average diameters > 18 mm, they were considered mature. Human chorionic gonadotropin (HCG) (10,000 IU) was injected, and the oocytes were collected via puncture 36 ~ 38 h later.

The embryos were cultured using a G-1 and G-2 sequential culture system (Vitrolife, Sweden) in K-MINK-1000 incubators (COOK, Australia) at 6% CO_2_, 5% O_2_, and 89% N_2_. The oocytes were fertilized at 3 ~ 4 h after retrieval using IVF or intracytoplasmic sperm injection (ICSI) depending on sperm quality and fertilization and then evaluated at 16 ~ 18 h after insemination for the presence of two pronuclei indicating normal embryos. Embryo morphology was examined on Day 3 (68 h after insemination), Day 5 (116 h after insemination), and Day 6 based on our laboratory evaluation criteria.

### Whole-exome sequencing and bioinformatic analysis

Genomic DNA was extracted from peripheral venous blood leukocytes from the patients and their parents. Whole-exome capture and sequencing were performed for the proband. Segregation analysis of the *TUBB8* variant was performed using Sanger sequencing on DNA samples of the three participating family members.

The *TUBB8* candidate variant was interpreted by following the American College of Medical Genetics and Genomics (ACMG)/Association for Molecular Pathology (AMP) clinical variant interpretation guidelines [26]. MEGA software was used to analyse conservation of the TUBB8 protein in primates. Based on homology modelling (Template: 6e88.1. B), SWISS-MODEL was used to predict the structure of the wild-type and mutant TUBB8 proteins, and the effect of the variant was analyzed using PYMOL software.

### In vitro experimental protocol

*TUBB8* wild-type (WT) or I4L mutated sequences were cloned and inserted into the pHAGE plasmid and verified by Sanger sequencing. These plasmids were transfected into HEK293T cells using Lipo2000 (Invitrogen, USA). At 48 h posttransfection, reverse transcription-quantitative polymerase chain reaction (RT‒qPCR) and western blotting (WB) were used to detect mRNA and protein expression. Details of the primers used are shown in Table 1.

Protein band signals were captured with Quantity One software. Data are shown as mean values (± SEM). Two-tailed Student’s *t* tests were used for statistical analysis.

**Table 1:** primers used in the experiments

	primer	primer sequences
vector construction	phage-TUBB8-SalI-wt-F	TGACGTCGACaATGAGGGAGATCGTGCTCAC
	phage-TUBB8-SalI-mut-F	TGACGTCGACaATGAGGGAGCTCGTGCTCAC
	phage-TUBB8-NotI-R	CGACGCGGCCGCTGGCCACCTCCTCCTCGGCAT
qPCR	TUBB8-phage-QPCR-F	ATCCACGCTGTTTTGACCTC
	TUBB8-phage-QPCR-R	CGATGGCATGTTCATCAGAG

Both WT and mutant *TUBB8* were transfected into HeLa cells. After 24 h, the transfected HeLa cells were fixed with 4% paraformaldehyde and permeabilized in PBS. The fixed cells were blocked with 10% goat serum and then incubated with Mouse anti FLAG-Tag (#3064, Dia-An Biotech, Wuhan, China) overnight at 4 ℃. The cells were then incubated at 37 ℃ with a secondary antibody (green; 1:1000) labeled with Alexa Fluor 488. A laser scanning confocal microscope FV1200 (Olympus, Japan) was used to visualize the cells with DAPI counterstaining.

## Results

### Patient characteristics

**Fig. 1:**
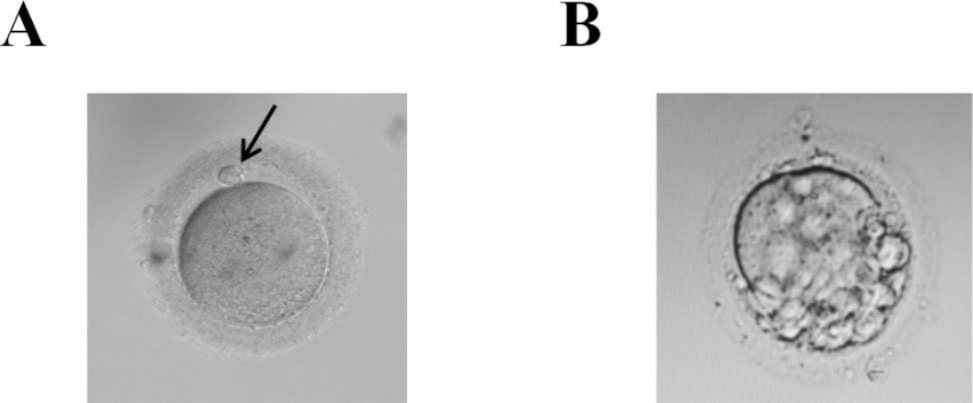
Morphology of oocytes retrieved from the proband compared with normal oocytes

The proband, a 27-year-old female, attended the Reproductive Medicine Center at the Second Affiliated Hospital of Zhengzhou University in December 2020. She was diagnosed with primary infertility. The patient had regular menstruation, normal levels of sex hormones, and a normal karyotype (46, XX). Her husband also had normal semen parameters (i.e., sperm concentration, motility, and sperm morphology) and karyotype. This couple underwent two failed intrauterine insemination (IUI) cycles and one failed IVF attempt. Twenty-three oocytes were retrieved, of which 10 were immature; the other 13 oocytes depicted abnormal morphology, such as multiple polar bodies or small fragments in the perivitelline space (PVS). No normal fertilized oocytes or cleavage embryos were observed (Table 2; Fig. 1).

The older sister of the proband was also diagnosed with primary infertility and underwent two failed IVF/ICSI cycles in another hospital five years prior. In December 2019, the sister attended our Reproductive Medicine Center and attempted her third IVF cycle. She also had regular menstruation, normal sex hormones, and karyotypes. In each cycle, oocytes of the MII stage could be observed, but no embryo was available for transfer. In the third cycle, we found three oocytes with abnormal morphology and multiple polar bodies or small fragments in PVS, which was not of concern (Table 2).

The process of controlled ovarian hyperstimulation showed that follicle growth and sex hormone levels were normal. There were no abnormalities in the incubators, reagents, and consumables. The patients denied any history of exposure to toxic or harmful substances. No other potential causes for abnormal oocytes were found.

**Table 2:** Clinical characteristics of the patients

Patients	Age (years)	Duration of infertility years)	IVF/ICSI cycles	COH protocol	Oocytes retrieved (n)	Immature oocytes (n)	MII stage (n)	Abnormal-morphology oocytes(n)	Fertilized oocytes(n)	Arrested embryos(n)
II-3 (proband)	27	7	1	Long-acting GnRH-a long protocol	23	10	0	13	0	0
II-1	29	9	3	1: Short-acting GnRH-a long protocol	9	7	2	Unknown	1	1
				2: Long-acting GnRH-a long protocol	18	12	6	Unknown	6	6
				3:Progestin-primed ovarian stimulation	8	2	6	3	1	1

A normal oocyte (A) and the oocyte from the proband (B) were separated from granulosa cells and examined by light microscopy. The normal metaphase II (MII) oocyte has a first polar body (black arrow ). The proband’s oocytes showed abnormal morphology, and there were multiple polar bodies or small fragments in perivitelline space (PVS).

### Genetic analysis revealed a *TUBB8* variant of uncertain significance

**Fig. 2:**
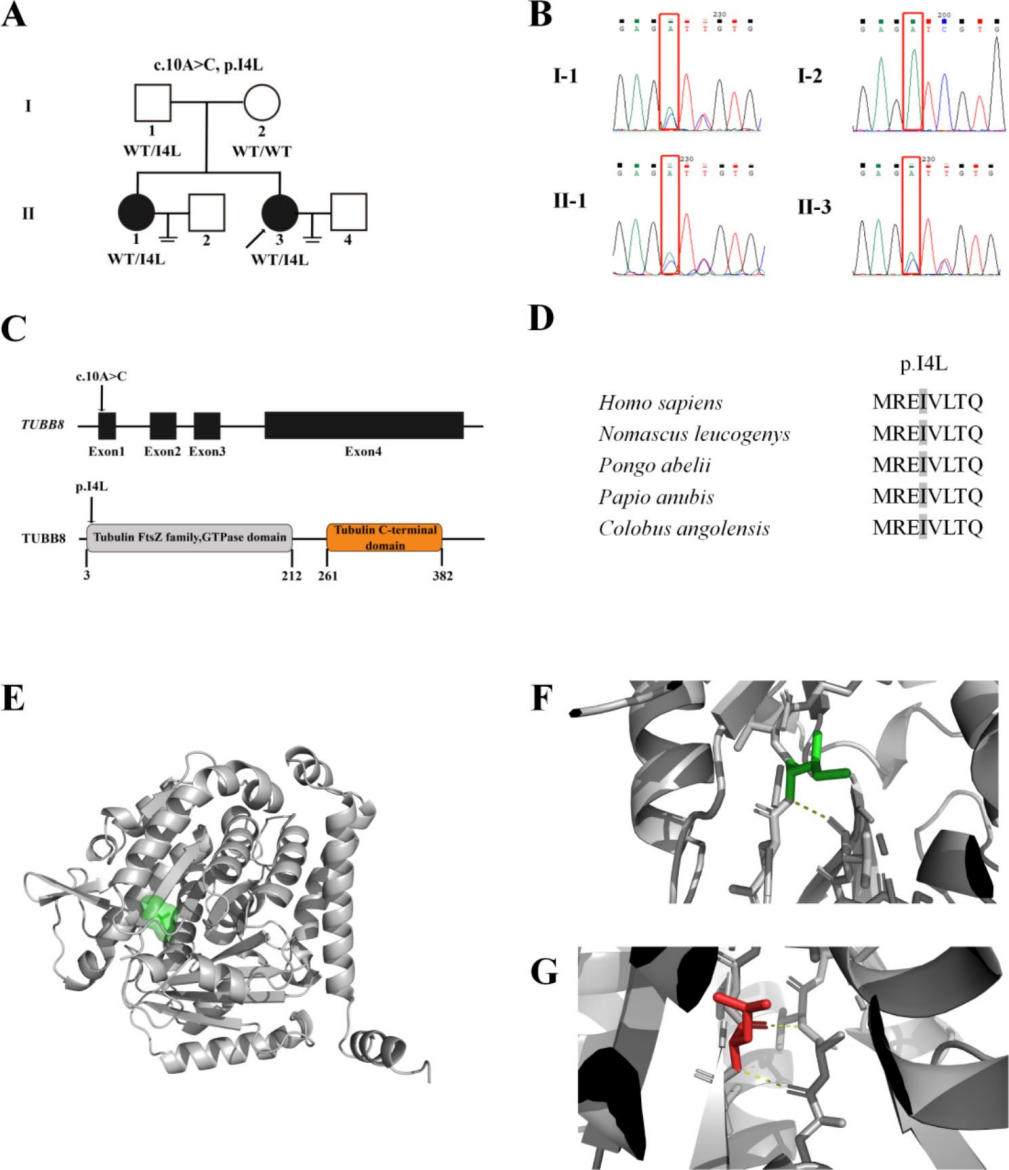
Genetic analysis of the *TUBB8* variant. (**A**) Family pedigree. Squares denote male family members, circles for female family members, solid symbols for affected family members, and open symbols for unaffected family members. Short double horizontal lines indicate that there were no offspring. The arrow indicates the proband. The I4L variant in *TUBB8* was inherited from the father. WT denotes wild type. (**B**) Sanger sequencing of the proband and her family members. (**C**) The position of altered alleles is shown on the gene structure of *TUBB8*, and the corresponding amino acid is indicated on the TUBB8 protein. (**D**) Conservation analysis of altered amino acids among five primate species. (**E**) The 3D model of TUBB8 protein and the location of the variant. (**F**) and (**G**) showed the protein conformation and hydrogen bond changes of WT (**F**) and mutant (**G**). Ile4 can form hydrogen bonds with Val49 in TUBB8 WT protein (**F**). In I4L mutant protein, the mutant Leu4 cannot form hydrogen bonds with Val49, and formed hydrogen bonds connections with Arg62 (bottom) and Val64 (top)

Analysis of the whole-exome sequencing data for the proband indicated a missense variant (c.10 A > C) in the *TUBB8* gene, which was confirmed using Sanger sequencing (Fig. 2B). Segregation analysis demonstrated that both sisters carried the same variant inherited from their father (Fig. 2A and B). No other gene variants related to oocyte maturation arrest or embryo development were detected.

This variant is located in exon 1, a part of the tubulin/FtsZ family in the GTPase domain (Fig. 2C). The amino acid in the mutated position is highly conserved across species (Fig. 2D). The variant is absent from the gnomAD database. Human Splicing Finder (HSF) predicted that this variant affects splicing through the alteration of exonic splicing silencer (ESS)/exonic splicing enhancer (ESE) binding sites. However, NetGene2 Server and Splice Site Prediction by Neural Network indicate that this variant has no apparent effect on mRNA splicing. This variant was interpreted as a variant of uncertain significance (VUS) (PM2-Supporting and PS4-Moderate) according to the ACMG/AMP variant classification framework (Table 3).

Three-dimensional (3D) model structures of the wild-type and mutant proteins were generated using Swiss-Model (Fig. 2E). The results showed that the mutant protein conformation was not significantly different from that of the WT protein. The hydrogen bonds at the variant had changed, which may affect the stability of the structural region (Figure 2F and G).

**Table 3:** Overview of the *TUBB8* variant

Gene	exon	cDNA change	Protein change	Mutation type	Genotype	gnomAD AF	ACMG standards
*TUBB8*	1	c.10 A > C	p.I4L	Missense	Het	0	VUS

### *TUBB8* I4L variant reduced mRNA and protein expression

**Fig. 3:**
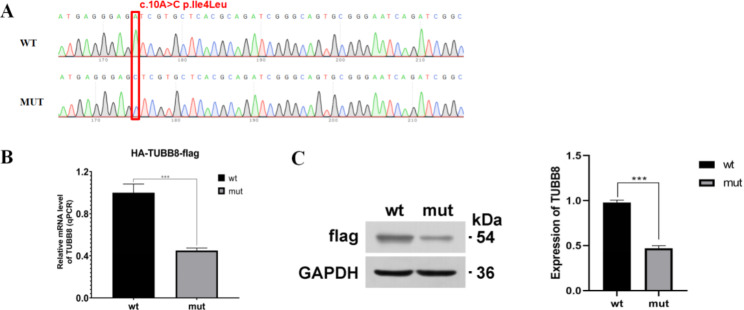
*TUBB8* expression analysis. (**A**) Sanger sequencing results revealed that the recombinant plasmid vector was successfully constructed. (**B**) RT‒qPCR results showed that mutant mRNA expression decreased. (**C**) WB results showed that the expression levels of mutant TUBB8 protein in HEK293T cells was decreased. ***P < 0.01

The results of RT‒qPCR analysis showed that mRNA expression of the mutant was 55% lower than that of WT. In the PHAGE vector, the WT and mutant proteins have a predicted molecular weight of approximately 54 kD. WB results revealed decreased expression of the mutated protein, consistent with the RT‒qPCR results (Fig. 3).

### *TUBB8* I4L variant did not change location of the protein

**Fig. 4:**
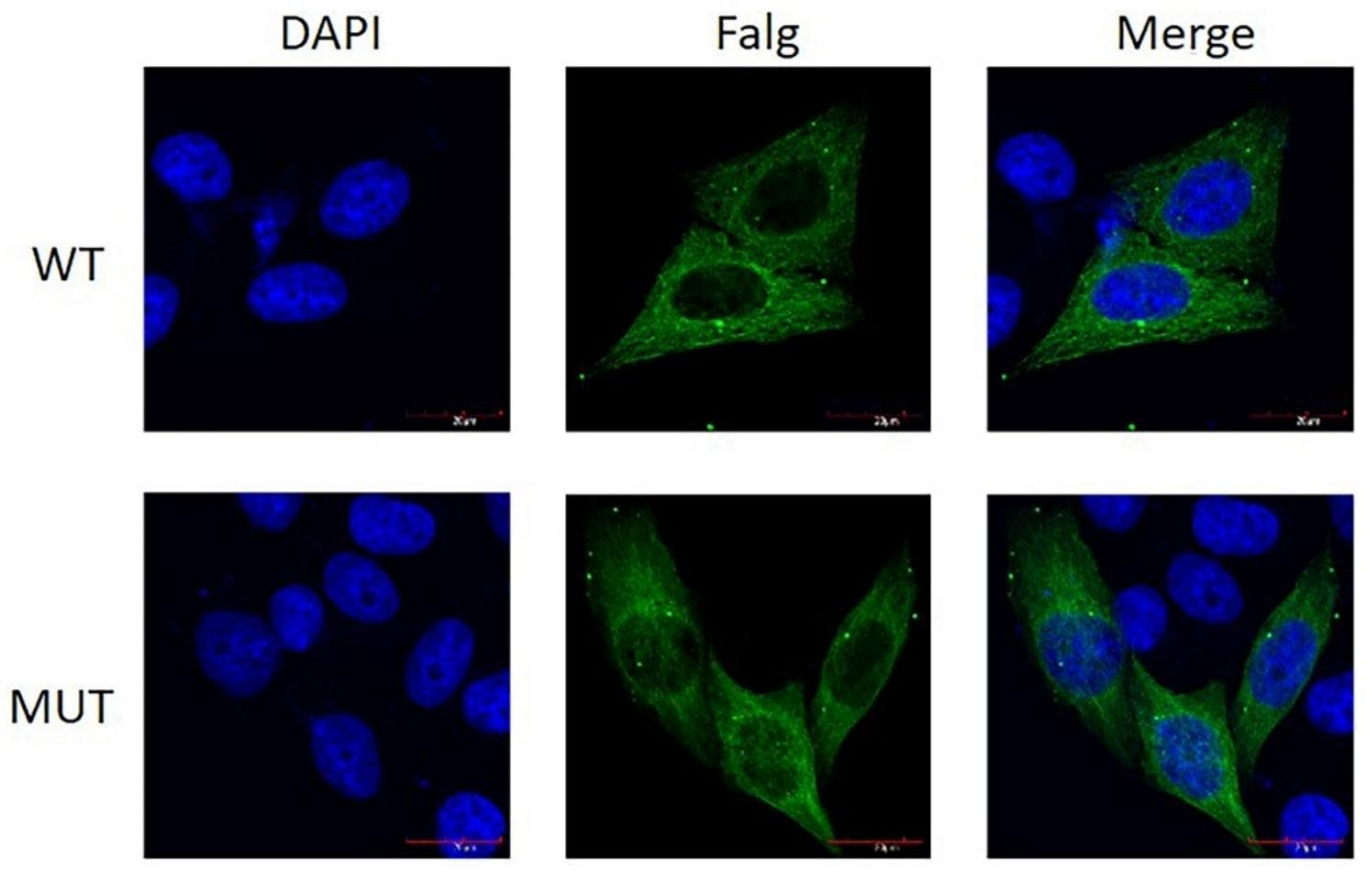
Immunofluorescent analysis of wild-type and mutant forms of TUBB8 in HeLa cells. The over-expression of TUBB8 of wild-type and mutant protein in HeLa cells was detected by Confocal microscopy. Mouse anti FLAG-Tag was used as primary antibody. ABflo® 488-conjugated Goat Anti-Mouse IgG (H + L) was used as secondary antibody (green). Nuclei were stained with DAPI (blue). The green staining in the merged images indicates the location of wild-type and mutant forms of TUBB8. The results showed that both wild-type and mutant TUBB8 were expressed in cytoplasm. Scale bar, 20 μm

Immunofluorescence experiments revealed that the wild-type and mutant fusion proteins were correctly located in the cytoskeleton and cytoplasm. This mutant did not change the localization of the protein (Fig. 4).

## Discussion

In this study, a heterozygous missense variant (c.10 A > C) in *TUBB8* was identified in a family of two sisters with primary infertility, with functional evidence of the pathogenicity of the variant. The variant was inherited from their father, who had no phenotype. This variant p.I4L was previously reported in patients with primary infertility; however, no typical oocyte maturation arrest was detected. The patient reported by Sha et al. [23] displayed multipronucleus (MPN) formation and 1PN in zygotes after IVF/ICSI; major MI arrest and minor 2PN arrest were found for the patient reported by Chen et al. [15]. Furthermore, Chen et al. [19] found two unrelated patients with p.I4L; mature oocytes could be retrieved, but the subsequent fertilization and cleavage rates were low. These reports demonstrate phenotypic variability in patients with the same variant. This heterogeneity of clinical phenotypes increases the difficulty of diagnosis.

TUBB encodes the β-tubulin protein and acts as a structural component of microtubules [27]. *TUBB8* is expressed specifically in human oocytes and embryos [13]. Variants in *TUBB8* can affect the assembly and function of microtubules, leading to spindle structure defects [13]. Males with *TUBB8* variants are fertile because *TUBB8* is not expressed in mature sperm, which also reflects the difference in meiosis and spindle formation between male and female germ cells [13]. There have been many studies on *TUBB8* with regard to differences in the phenotypic diversity of patients with variants. Pathogenic variants in *TUBB8* have been associated with oocyte maturation arrest, oocytes with a large polar body, abnormal fertilization, fertilization failure, no cleavage, early embryonic arrest, and embryonic implantation failure [13–25]. The main reason is that the effects of these variants on protein function may differ in severity. Some variants have a significant negative effect, causing severe spindle defects or absence as well as characteristic oocyte maturation arrest [15, 19]. However, oocytes carrying some variants can show visible spindles with abnormal morphology. Such variants were associated with a relatively less severe clinical phenotype, as oocytes failed to become fertilized, zygotes failed to cleave, and early embryonic development was arrested [15, 19]. This study found that phenotypes also differ between individuals or siblings with the same variants.

This variant, p.I4L, is located within the GTPase domain of the tubulin/FtsZ family. The tubulin/FtsZ protein family plays crucial roles in cellular processes such as cell division and establishment of the cytoskeleton in eukaryotes [28]. Misato and Dml1p are derived from an ancestral tubulin-like protein and possess regions that share similarity with members of a GTPase family, including eukaryotic tubulin and prokaryotic FtsZ [29]. In Drosophila, variants in the misato gene have been demonstrated to impede kinetochore-driven microtubule growth, forming monopolar spindles and causing larval lethality [30]. *Saccharomyces cerevisiae* embedded *TUBB8* variants were not viable [13]. These results indicate that MT formation is an essential process for proper spindle assembly.

To further understand the functional consequences of this variant, HEK293T cells were transfected with wild-type and mutant *TUBB8*. To our knowledge, this study presents the first confirmation that the variant p.I4L reduces the level of *TUBB8* mRNA and protein. A reduction in tubulin levels might disrupt the integrity of the microtubule network. However, subsequent immunofluorescence revealed that this mutant does not change protein localization. As previously reported, these results indicate that other β-tubulin isotypes also contribute to spindle structure [19]. Heterogeneity in clinical phenotype may be due to the cumulative effect of variation on protein function. A severe phenotype will appear only when the abnormal protein reaches a critical threshold [13]. If the microtubule network is not severely impaired, different degrees of the clinical phenotype occur in individual patients with the same variant.

Currently, no cure exists for oocyte maturation arrest and abnormal embryonic development caused by gene defects. Although wild-type cRNA or miRNA injection can reverse phenotype in mouse or human oocytes [22], more animal models and clinical research are needed to assess the effectiveness and safety before clinical application. It is difficult to consider the injection dose, and most studies are limited to specific variants; consequently, experimental confirmation is required for other patients.

Clinically, once a diagnosis of infertility caused by gene defects is made, the patient would be advised to receive egg donation, and patients could not have genetic offspring. Therefore, such a diagnosis must be made with great caution, and appropriate validation research is warranted to confirm the relationship between the pathogenic variant and the disease. In this study, a small number of mature oocytes could be retrieved from the older sister, but genetic analysis could not provide a definite conclusion. It is predicted that this variant might not obviously affect mRNA splicing or protein 3D structure. In accordance with the ACMG/AMP classification, this variant has been categorized as “VUS”. To obtain additional confirmation, in vitro experiments were conducted to ascertain the detrimental impact of the variant on *TUBB8*. The results of functional assays suggest that this variant could be reclassified as “likely pathogenic”, emphasizing the significance of functional validation in the determination of pathogenicity.

In conclusion, this study demonstrates phenotypic variability in female siblings with the same *TUBB8* variant. It was confirmed that the mutant p.I4L decreases the level of *TUBB8* mRNA and protein but does not change the protein localization. Consequently, a definitive genetic diagnosis can help guide treatment for such patients.

### Electronic supplementary material

Below is the link to the electronic supplementary material.

Supplementary Material 1

Supplementary Material 2

## Data Availability

The sequence data during the current study are available in the NCBI SRA under accession number PRJNA909219 (https://www.ncbi.nlm.nih.gov/sra/?term=PRJNA909219). The other datasets generated and analyzed in this study are included in this manuscript.

## Abbreviations

*TUBB8*: Tubulin beta-8
ART: Assisted reproductive technology
IVF: In vitro fertilization
MTs: Microtubules
GnRH: Gonadotropin-releasing hormone
HCG: Human chorionic gonadotropin
ICSI: Intracytoplasmic sperm injection
COH: Controlled ovarian hyperstimulation
ACMG: American College of Medical Genetics and Genomics
AMP: Association for Molecular Pathology
WT: Wild-type
RT‒qPCR: Reverse transcription-quantitative polymerase chain reaction
WB: Western blotting
PVS: Perivitelline space
HSF: Human Splicing Finder
ESS: Exonic splicing silencer
ESE: Exonic splicing enhancer
MPN: Multipronucleus
