# Supplementary material for: Phenotypic variability in two female siblings with oocyte maturation arrest due to a TUBB8 variant
Source: BMC Med Genomics. 2023 Oct 30;16:271. doi: 10.1186/s12920-023-01712-7 (PMC10614405; doi:10.1186/s12920-023-01712-7)
Supplement: Supplementary file 1 — Supplementary Material 1 [file 12920_2023_1712_MOESM1_ESM.docx]

***DNA Library Preparation***

For exome sequencing, we fragmented 1–3 μg of genomic DNA, extracted from each sample, to an average size of 180 bp with a Bioruptorsonicator (Diagenode). Paired-end sequencing libraries then were prepared using a DNA sample prep reagent set 1 (NEBNext). Library preparation included end repair, adapter ligation and PCR enrichment, and was carried out as recommended by Illumina protocols.

***Targeted genes enrichment and sequencing***

The amplified DNA was captured use GenCap Deafness capture kit (MyGenostics GenCap Enrichment technologies). The DNA probes were designed to tile along the exon regions and the known non-exon pathogenic region of human genes. The capture experiment was conducted according to manufacturer’s protocol. In brief, 1μg DNA library was mixed with Buffer BL and GenCap gene panel probe (MyGenostics, Beijing, China), heated at 95°C for 7 min and 65°C for 2 min on a PCR machine; 23μl of the 65°C prewarmed Buffer HY (MyGenostics Inc，Beijing，China) was then added to the mix, and the mixture was held at 65°C with PCR lid heat on for 22 hours for hybridization. 50 μl MyOne beads (Life Technology) was washed in 500μL 1X binding buffer for 3 times and resuspended in 80μl 1X binding buffer. 64 μl 2X binding buffer was added to the hybrid mix, and transferred to the tube with 80μl MyOne beads. The mix was rotated for 1 hour on a rotator. The beads were then washed with WB1 buffer at room temperature for 15 minutes once and WB3 buffer at 65°C for 15 minutes three times. The bound DNA was then eluted with Buffer Elute. The eluted DNA was finally amplified for 15 cycles using the following program: 98°C for 30 s (1 cycle); 98°C for 25 s, 65°C for 30 s, 72°C for 30 s (15 cycles); 72°C for 5 min (1 cycle). The PCR product was purified using SPRI beads (Beckman Coulter) according to manufacturer’s protocol. The enrichment libraries were sequenced on Illumina HiSeq X ten sequencer for paired read 150bp.

***Bioinformatics analysis***

After sequencing, the raw data were saved as a FASTQ format, then followed the bioinformatics analysis: First, Illumina sequencing adapters and low quality reads (<80bp) were filtered by cutadapt. After quality control, the clean reads were mapped to the UCSC hg19 human reference genome using BWA. Duplicated reads were removed using picard tools and mapping reads were used for variation detection. Second, the variants of SNP and InDel were detected by GATK HaplotypeCaller, then using GATK Variant Filtration to filter variant, the filtered standard as follows: a) variants with mapping qualities < 30; b) the Total Mapping Quality Zero Reads < 4; c) approximate read depth < 5; d)QUAL<50.0; e) phred-scaled p-value using Fisher's exact test to detect strand bias > 10.0. After above two steps, the data would be transformed to VCF format, variants were further annotated by ANNOVAR and associated with multiple databases, such as,1000 genome, ESP6500, dbSNP , EXAC, Inhouse (MyGenostics), HGMD, and predicted by SIFT, PolyPhen-2, MutationTaster, GERP++.

***Variants Selected***

In this course, five steps using to select the potential pathogenic mutations in downstream analysis: (i) Mutation reads should be more than 5, mutation ration should be no less than 30%; (ii) Removing the mutation, the frequency of which showed more than 5% in 1000g, ESP6500 and Inhouse database; (iii) If the mutations existed in InNormal database (MyGenostics), then dropped; (iv) Removing the synonymous. (v) After (i),(ii),(iii), if the mutations were synonymous and they were reported in HGMD, left them. When finished above jobs, the mutations which were left should be the pathogenic mutations.

***Software and database***

1. BWA：<http://bio-bwa.sourceforge.net/>

Li H, Durbin R. Fast and accurate short read alignment with Burrows–Wheeler transform. Bioinformatics.2009; 25: 1754–1760.

1. Picard：<http://broadinstitute.github.io/picard/>
2. Cutadapt：http://cutadapt.readthedocs.io/en/stable/
3. GATK：<https://software.broadinstitute.org/gatk/>
4. ANNOVAR：<http://annovar.openbioinformatics.org/en/latest/>

Wang K, Li M, Hakonarson H. ANNOVAR: functional annotation of genetic variants from high-throughputsequencing data. Nucleic Acids Res. 2010; 38: e164.

1. 1000 genome：<http://www.1000genomes.or/>
2. EVS：<http://evs.gs.washington.edu/EVS>
3. dbSNP ：<http://www.ncbi.nlm.nih.gov/projects/SNP/>
4. EXAC：<http://exac.broadinstitute.org/>
5. HGMD：<http://www.biobase-international.com/product/hgmd>
6. SIFT：<http://sift.jcvi.org/>
7. PolyPhen-2：<http://genetics.bwh.harvard.edu/pph2/>
8. MutationTaster：<http://www.mutationtaster.org/>
9. GERP++：<http://mendel.stanford.edu/SidowLab/downloads/gerp/index.html>
10. SPIDEX：<http://www.deepgenomics.com/spidex>
