# Supplementary figures and images for: Phenotypic variability in two female siblings with oocyte maturation arrest due to a TUBB8 variant
Source: BMC Med Genomics. 2023 Oct 30;16:271. doi: 10.1186/s12920-023-01712-7 (PMC10614405; doi:10.1186/s12920-023-01712-7)

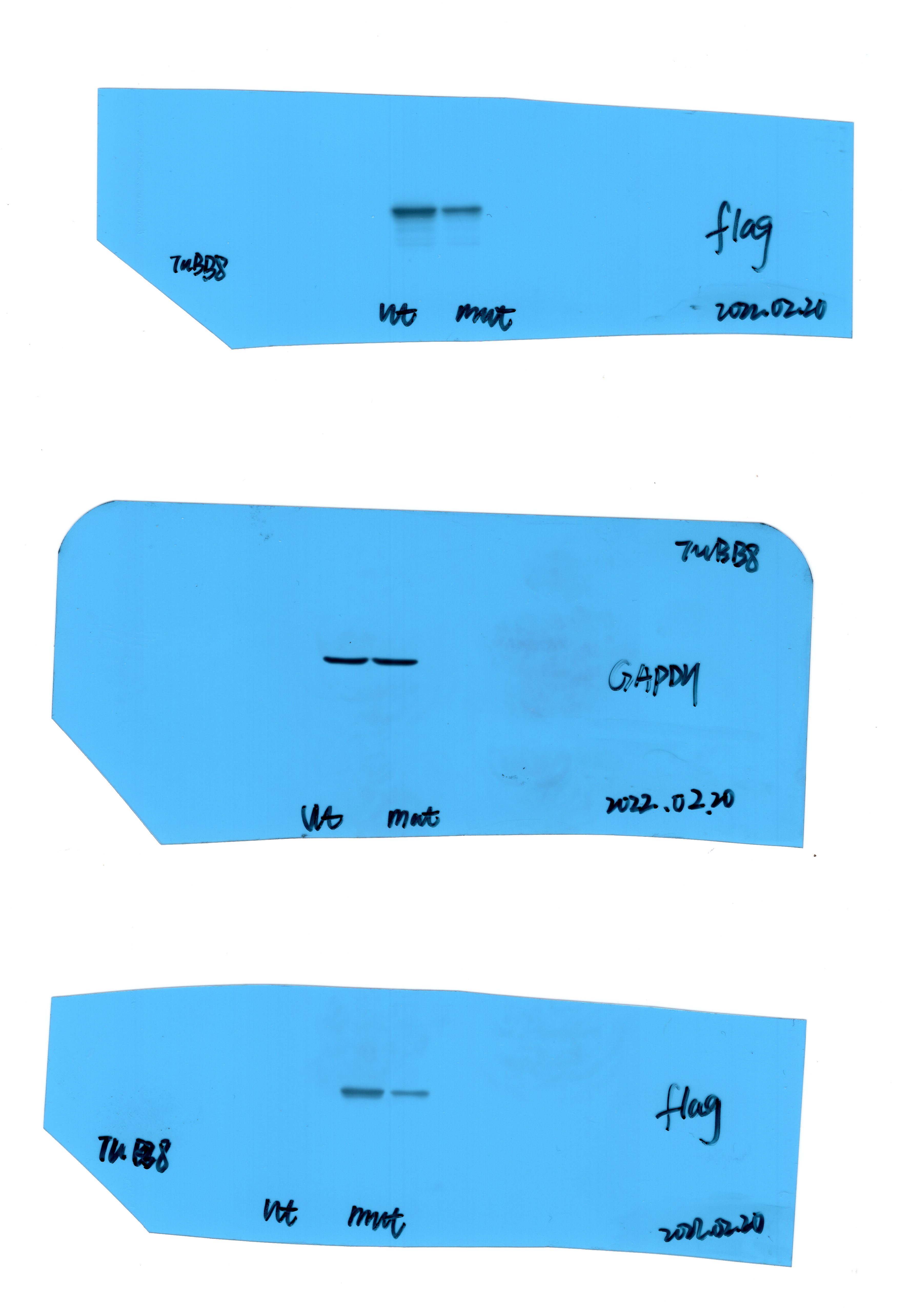

Supplement: Supplementary file 2 — Supplementary Material 2 [file 12920_2023_1712_MOESM2_ESM.jpg]
